# Supplementary material for: Identification of photoperiod-induced specific miRNAs in the adrenal glands of Sunite sheep (Ovis aries)
Source: Front Vet Sci. 2022 Jul 22;9:888207. doi: 10.3389/fvets.2022.888207 (PMC9354845; doi:10.3389/fvets.2022.888207)
Supplement: Supplementary file 1 [file Data_Sheet_1.ZIP › Supplementary file/miRDeep2 file.zip!/miRDeep2 file/Know_Structure/SPLP42A2/oar-mir-27a.pdf]

miRBase precursor : oar-mir-27a
Total read count : 356755
oar-miR-27a read count : 356385
remaining reads : 370

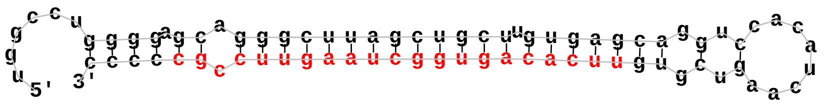

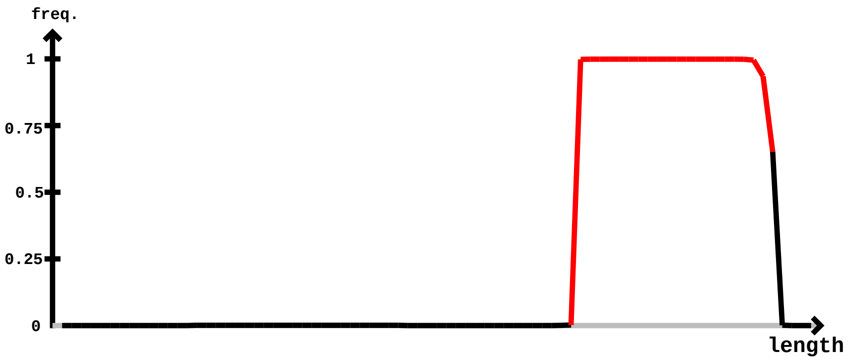


```
                                                   oar-miR-27a
5'- uggccuggggagcagggcuuagcugcuugugagcagguccacaucaagucguguucacaguggcuaaguuccgccccc  -3'   exp
    ......((((.((.(((((((((((((.(((((((.(.(........).).))))))))))))))))))))).))))))   reads   mm   sample
    .............agggcuuagcugcuugug...............................................   2       0    seq
    .............agggcuuagcugcuuguga..............................................   1       0    seq
    .............agggcuuagcugcuugugag.............................................   2       0    seq
    .............agggcuuagcugcuugugagc............................................   54      0    seq
    .............agggcuuagcugcuugugagca...........................................   298     0    seq
    .............agggcuuagcugcuugugagcagg.........................................   5       0    seq
    .............agggcuuagcugcuugugagcaggu........................................   5       0    seq
    .............agggcuuagcugcuugugagcagguc.......................................   1       0    seq
    ..............gggcuuagcugcuugugagca...........................................   1       0    seq
    ................................................ucguguucacaguggcuaagu.........   1       0    seq
    ...................................................uguucacaguggcuaaguu........   1       0    seq
    ...................................................uguucacaguggcuaaguuc.......   8       0    seq
    ...................................................uguucacaguggcuaaguucU......   6       1    seq
    ...................................................uguucacaguggcuaaguucc......   18      0    seq
    ...................................................uguucacaguggcuaaguucUg.....   2       1    seq
    ...................................................uguucacaguggcuaaguuccg.....   209     0    seq
    ...................................................uguucacaguggcuaaguucUgc....   1       1    seq
    ...................................................uguucacaguggcuaaguuccgc....   15      0    seq
    ...................................................uguucacaguggcuaaguuccgcc...   1       0    seq
    ....................................................guucacaguggcuaaguuc.......   2       0    seq
    ....................................................guucacaguggcuaaguucc......   2       0    seq
    ....................................................guucacaguggcuaaguucU......   35      1    seq
    ....................................................guucacaguggcuaaguuccg.....   1       0    seq
    ....................................................guucacaguggcuaaguucUg.....   115     1    seq
    ....................................................guucacaguggcuaaguuccgc....   2       0    seq
    ....................................................guucacaguggcuaaguucUgc....   369     1    seq
    .....................................................uucacaguggcuaaguG........   6       1    seq
    .....................................................uCcacaguggcuaaguu........   2       1    seq
    .....................................................uucUcaguggcuaaguu........   1       1    seq
    .....................................................uucacaguggcuaaguu........   104     0    seq
    .....................................................uucacaguAgcuaaguu........   1       1    seq
    .....................................................uucacaguggcuaaguuG.......   8       1    seq
    .....................................................uucacaguggcuaaguuc.......   1137    0    seq
    .....................................................uucacaguAgcuaaguuc.......   1       1    seq
    .....................................................uucacaguggcuaaguucU......   17950   1    seq
```

oar-miR-27a

```
uggccuggggagcagggcuuagcugcuugugagcagguccacaucaagucguguucacaguggcuaaguuccgccccc
.....................................................uucacaguggcuaaguucc......     3542      0          seq
.....................................................uucacaguggcuaaguuccg.....     54766     0          seq
.....................................................uucacaguggcuaaguuccA.....     11730     1          seq
.....................................................uucacaguggcuaaguucUg.....     34078     1          seq
.....................................................uucacaguggcuaaguuccgc....     7249      0          seq
.....................................................uucacaguggcuaaguucUgc....     224221    1          seq
.....................................................uucacaguggcuaaguuccgcc...     455       0          seq
.....................................................uucacaguggcuaaguuccgccc..     3         0          seq
......................................................ucacaguggcuaaguuc.......     2         0          seq
......................................................ucacaguggcuaaguucc......     2         0          seq
......................................................ucacaguggcuaaguucU......     25        1          seq
......................................................ucacaguggcuaaguuccg.....     39        0          seq
......................................................ucacaguggcuaaguuccA.....     8         1          seq
......................................................ucacaguggcuaaguucUg.....     22        1          seq
......................................................ucacaguggcuaaguuccgc....     6         0          seq
......................................................ucacaguggcuaaguucUgc....     189       1          seq
.......................................................cacaguggcuaaguucc......     1         0          seq
.......................................................cacaguggcuaaguucU......     1         1          seq
.......................................................cacaguggcuaaguuccg.....     5         0          seq
.......................................................cacaguggcuaaguucUg.....     6         1          seq
.......................................................cacaguggcuaaguucUgc....     27        1          seq
........................................................acaguggcuaaguuccg.....     2         0          seq
........................................................acaguggcuaaguucUg.....     1         1          seq
........................................................acaguggcuaaguuccgc....     1         0          seq
........................................................acaguggcuaaguucUgc....     5         1          seq
.........................................................caguggcuaaguucUgc....     2         1          seq
```
